# A field-friendly alternative to freeze-drying faeces for glucocorticoid metabolite analyses of African wild dogs (*Lycaon pictus*)

**DOI:** 10.1016/j.mex.2022.101623

**Published:** 2022-01-16

**Authors:** Gabriella Postiglione, Pier Attilio Accorsi, Andre Ganswindt, Bruce Crossey

**Affiliations:** a Department of Veterinary Medical Sciences, Alma Mater Studiorum, University of Bologna, Italy; b Mammal Research Institute (MRI), Department of Zoology and Entomology, Faculty of Natural and Agricultural Sciences, University of Pretoria, South Africa; c Endocrine Research Laboratory, Faculty of Natural and Agricultural Sciences, University of Pretoria, South Africa; d Centre for Veterinary Wildlife Studies, Faculty of Veterinary Science, University of Pretoria, South Africa

**Keywords:** Non-invasive, Stress, Steroids, Wild dog faeces, Lyophilisation

## Abstract

Endocrine studies using faeces as hormone matrix have become increasingly popular to examine adrenocortical activity in wildlife. A prerequisite for this approach is to minimize alteration of faecal glucocorticoid metabolite (fGCM) composition post-defecation. This is done by freezing the collected material as soon as possible after collection, and removing moisture from the frozen faecal samples afterwards (usually by freeze-drying). In remote areas, freeze-drying opportunities are often limited, and in the case of the African wild dog (*Lycaon pictus*), established assays revealed that fGCM concentrations remain comparable for only ∼24h post-defaecation. In the present study, three cost-effective drying treatments (exposure to sunlight, placement in a solar oven, and use of a food dehydrator) were investigated as alternatives to the golden standard of freeze-drying faeces.

• In comparison to freeze-dried material, African wild dog faecal samples dried through sunlight exposure, a solar oven, and use of a food dehydrator revealed no significant differences in respective fGCM concentrations measured.
• A food dehydrator would be the preferable option to dry African wild dog faeces if limited electrical supply is available. This technique dries faeces the fastest, and negates any reliance on weather conditions.

## Graphical abstract

**Figure:**
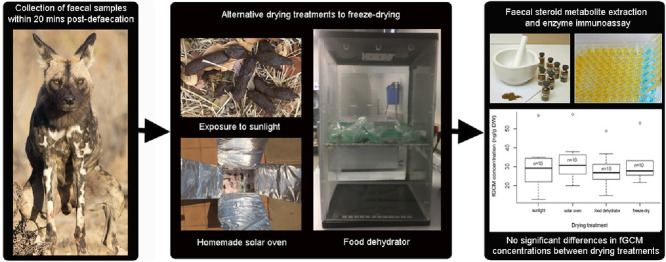

Specifications table

**Table:** 

Subject Area:	Agricultural and Biological Sciences
More specific subject area:	Zoology
Method name:	Drying of faecal samples for glucocorticoid metabolite extraction and quantification.
Name and reference of original method:	B. Crossey, A. Ganswindt, C.T. Chimimba. Faecal glucocorticoid metabolite concentrations and their alteration post-defaecation in African wild dogs *Lycaon pictus* from South Africa, Wildl. Biol. (2018).
Resource availability:	N/A

## Background information

Non-invasive hormone monitoring using faeces as matrix has become a widely-used approach to examine factors that animals may perceive as stressors [1]. This is because faecal sampling, as opposed to an invasive approach (like blood collection), facilitates safe, feedback-free sample collection, with animals not usually disturbed during the process [1]. Quantifying faecal steroids also allows for the assessment of a more cumulative hormone signal [2], as faecal hormone metabolite concentrations represent an accumulation of the fluctuating secretions and eliminations of hormones circulating in the blood [3,4].

One of the biggest challenges associated with faecal steroid analyses is the stability of faecal hormone metabolite concentrations post-defecation. Glucocorticoids are metabolized in the liver and gut prior to excretion, and post-defaecation, bacterial enzymes present in the faeces continue this process [5]. Freezing of the faeces halts the action of bacterial enzymes, as does the removal of moisture from the faeces (usually achieved by freeze-drying) [6]. As a result, standardized collection procedures, often involving the freezing of faecal material within a defined period post-defaecation have been developed to ensure the comparability of determined faecal steroid concentrations [2].

Under field conditions, where the freezing of samples is often delayed, or cannot be maintained over a longer period, this approach becomes more challenging. This is particularly true for studies focusing on wide-ranging species, such as the African wild dog (*Lycaon pictus*), where sampling is often conducted at field sites with a limited electrical supply [see [7], [8], [9]]. In many such cases, a sporadic electrical supply provides a meaningful challenge to keep samples frozen over a prolonged period, including during the subsequent transport to an analytical laboratory. Even in cases where freezing, and the subsequent maintenance of frozen samples is possible, freeze-drying (lyophilisation) is considered the optimal method to remove moisture from frozen faecal samples. This process can be both time-consuming and costly, as freeze-dryers are expensive pieces of equipment that are not always readily accessible, and are impractical for use in the field.

## Faecal sampling

Faecal samples were opportunistically collected from free-ranging African wild dogs in the Limpopo Lipadi Private Game and Wilderness Reserve (n=5) in south eastern Botswana, from October 2017- August 2018), as well as in the Khamab Nature Reserve (n=5), northern South Africa (from July-September 2018). All faecal material was collected from known individuals within 20 minutes after observing defaecation events. In each case, the entire dropping was collected, placed into a sealable plastic bag, immediately placed on ice, and frozen within two hours at -20°C until further processing. This study was conducted with the approval of the Animal Welfare Committee (COBA) – University of Bologna (Protocol No. 0003606); the Ministry of Environment, Natural Resources Conservation and Tourism, Republic of Botswana (Research Permit reference: ENT 8/36/4 II (4)); as well as per the terms of Section 20 of the Animal Diseases Act, 1984 (ACT NO 35 of 1984), issued by the Department of Agriculture, Forestry and Fisheries, Republic of South Africa (Ref. 12/11/1/1/20 – 847).

## Drying treatments

**Fig. 1:**
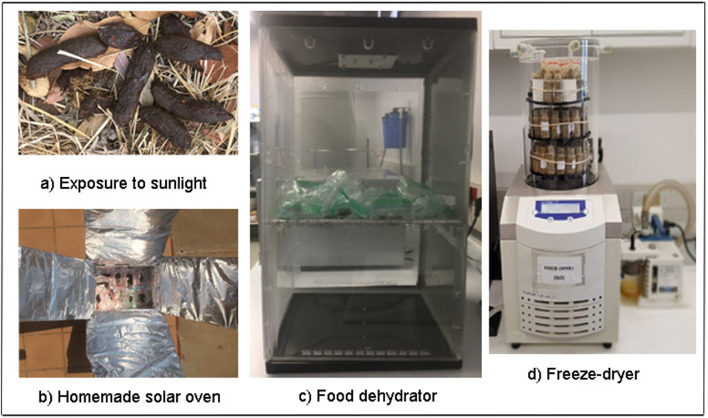
The four different drying treatments used to remove moisture from African wild dog (*Lycaon pictus*) faecal samples.

All collected samples were defrosted and, individually, thoroughly mixed by hand over a cool surface. Mixed faecal material from each individual African wild dog was then divided into a subset of four samples to be tested across all drying treatments; these included: i) direct exposure to the sun (Fig. 1a); ii) a homemade solar oven (a cardboard box covered in tin foil; Fig. 1b); a food dehydrator (Model: K002, Mellerware, Johannesburg, South Africa) which makes use of a B22D 40W halogen lamp and circulating fan to provide continuous airflow (Fig. 1c); and iv) freeze-drying (Model: ALPHA 1-2 LD plus, Christ, Osterode, Germany; Fig. 1d).

Samples dried with the three alternative methods to freeze-drying were weighed every four hours from 07:00 to 19:00, and were considered dry when subsequent masses differed by < 0.01 g. Within each alternative treatment, samples were also rotated every 4h between 07:00 and 19:00 to ensure similar exposure to heat for each sample-aliquot. Temperature was measured every 4h, and ranged between 14-43°C for the samples exposed to sunlight, 14-37°C inside the solar oven, and 29-32°C inside the food dehydrator. Once dried, samples were re-sealed in their bags and stored at room temperature (20-25°C) until assayed.

## Enzyme immunoassay (EIA)

Dried faeces were pulverized, and sifted with a metal strainer to separate faecal powder from undigested material [10]. Following standardized procedures [11], [12], [13], between 0.050 and 0.055g faecal powder was extracted using 3 ml of 80% ethanol in water by vortexing for 15 min. Supernatant, obtained after centrifuging at 1 500 g for 10 min, was transferred into sealed micro-centrifuge tubes and stored at −20°C until hormone analysis [11]. Steroid extracts were measured for fGCM concentrations using an enzyme immunoassay (EIA) established for African wild dogs by ACTH challenge [13]. This competitive EIA utilizes antibodies against cortisol-3-CMO:BSA and a cortisol-3-CMO-DADOO-biotin label. Further details relating to assay components, antibody cross-reactivities, and other assay characteristics are provided by Palme and Möstl [14]. Sensitivity of the assay was 0.6 ng/g dry weight (DW). Intra- and inter-assay coefficients of variation (CV), determined by repeated measurements of high and low quality controls were 5.67% and 6.90% (Intra-assay CV), and 9.39% and 13.49% (Inter-assay CV), respectively. Steroid extraction and assays were conducted in the Endocrine Research Laboratory, University of Pretoria, South Africa, and followed published protocols [12].

## Alternatives to lyophilisation

**Fig. 2:**
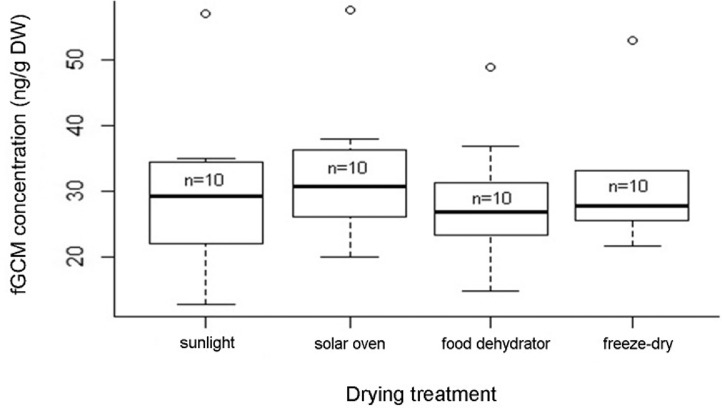
African wild dog (*Lycaon pictus*) fGCM concentrations (median, lower and upper quartiles are represented by boxes, range is represented by dotted lines and whiskers, and open dots indicate outlier data points) for sub samples tested across different drying treatments.

A non-parametric Kruskal-Wallis Analysis of Variance (ANOVA), conducted using algorithms in *R* (www.r-project.org), with the use of the *R Studio* interface (www.r-project.org), was run to test for differences in fGCM concentrations across the different drying treatments. No significant differences in fGCM concentrations were found between the different treatments (*H*_3_= 1.75; *n=*40; *P*>0.50; Fig. 2). This bodes well for the inclusion of fGCM analyses to conservation and physiology studies conducted on African wild dogs in areas where a limited electrical supply is available. The results of this study provide more options for researchers facing logistical challenges and time constraints in the field, as an applicable drying treatment for faeces can now be selected based on the availability of resources and applied on-site.

These results demonstrate that African wild dog faeces can be dried using methods other than the logistically challenging process of freeze-drying, without compromising fGCM analysis. The use of a food dehydrator, in particular, provides a cost-friendly alternative to freeze-drying that would be preferable when a limited power supply is available. Using a food dehydrator resulted in the shortest drying time of the alternatives to freeze-drying (with samples drying in 71h, compared to 125h in the solar oven, and 135h when exposed to the sun). This, while also negating any reliance on sunny and clear weather conditions, and providing protection for the samples against insects, birds and monkeys, which often act as pests in the field. These findings are important, as results from Crossey et al. [8] indicate that wild dog fGCM concentrations continue to be affected by bacterial enzyme activity after samples have been frozen and then defrosted. In this study, measured wild dog fGCM concentrations reached an increase of ∼155% above the initial mean at 96 hours post-defrosting. This indicates that the initial freezing of faeces is not sufficient to permanently inhibit bacterial enzyme activity, and further processing is required to completely remove all moisture from a sample.

It should be noted that these results apply to fGCM quantification utilizing the EIA indicated above, and the applicability of alternative drying methods may vary across a range of steroid extraction protocols and EIAs. Different EIAs have been known to reflect similarly distinct increases and decreases in fGCM concentrations when measuring different fGCMs (e.g. fGCMs with a 5β-3α-ol-11-one structure *versus* 11, 17 dioxandrostanes in sheep (*Ovis aries*) [15]). This variation may also occur when examining different steroid classes in the same faecal sample [16]. Future research should thus focus on the efficacy of these cost-effective alternatives to freeze-drying for other species and different steroid classes.

## Conclusion

The use of a food dehydrator, homemade solar oven, or exposure to sunlight provides viable and cost-effective alternatives to freeze-drying for African wild dog faeces when measuring fGCM concentrations. These alternative drying methods can assist researchers to include fGCM analyses in their studies without the need for costly and logistically challenging freeze-drying techniques, thus promoting the use of this non-invasive means of hormone monitoring as a tool in conservation and physiological studies.

## References

[1] Ganswindt A., Brown J.L., Freeman E.W., Kouba A.J., Penfold L.M., Santymire R.M., Vick M.M., Wielebnowski N., Willis E.L., Milnes M.R. (2012). International Society for Wildlife Endocrinology: the future of endocrine measures for reproductive science, animal welfare and conservation biology. Biol. Lett..

[2] Hulsman A., Dalerum F., Ganswindt A., Muenscher S., Bertschinger H.J., Paris M. (2011). Non-invasive monitoring of glucocorticoid metabolites in brown hyaena (*Hyaena brunnea*) feces. Zoo Biol.

[3] Möstl E., Rettenbacher S., Palme R (2005). Measurement of corticosterone metabolites in birds' droppings: an analytical approach. Ann. N. Y. Acad. Sci..

[4] Touma C., Palme R. (2005). Measuring fecal glucocorticoid metabolites in mammals and birds. Ann. N.Y. Acad Sci..

[5] Palme R. (2005). Measuring of fecal steroids: guidelines for practical Application. Ann. N.Y. Acad. Sci..

[6] Washburn B.E., Millspaugh J.J. (2002). Effects of simulated environmental conditions on glucocorticoid metabolite measurements in white-tailed deer feces. Gen. Compar. Endocrinol..

[7] Creel S. (2005). Dominance, aggression and glucocorticoid levels in social carnivores. J. Mammal..

[8] Crossey B., Ganswindt A., Chimimba C.T. (2018). Faecal glucocorticoid metabolite concentrations and their alteration post-defaecation in African wild dogs *Lycaon pictus* from South Africa. Wildl. Biol..

[9] Crossey B., Chimimba C.T., du Plessis C., Hall G., Ganswindt A. (2020). Using faecal glucocorticoid metabolite analyses to elucidate stressors of African wild dogs *Lycaon pictus* from South Africa. Wildl Biol.

[10] Fieß M., Heistermann M., Hodges J.K. (1999). Patterns of urinary and fecal steroid excretion during the ovarian cycle and pregnancy in the African elephant (*Loxodonta africana*). Gen. Compar. Endocrinol..

[11] A.Ganswindt S.Münscher, Henley M., Palme R., Thompson P., Bertschinger H. (2010). Concentrations of faecal glucocorticoid metabolites in physically injured free-ranging African elephants *Loxodonta africana*. Wildl. Biol..

[12] Ganswindt A., Heistermann M., Borragan S., Hodges J.K. (2002). Assessment of testicular endocrine function in captive African elephants by measurement of urinary and fecal androgens. Zoo Biol.

[13] Vlamings B.H.A.C. (2011).

[14] Palme R., Möstl E. (1997). Measurement of cortisol metabolites in faeces of sheep as a parameter of cortisol concentration in blood. Z. Saeugetierkunde.

[15] Lexen E., El-Bahr S.M., Sommerfeld-Stur I., Palme R., Möstl E. (2011). Monitoring the adrenocortical response to disturbances in sheep by measuring glucocorticoid metabolites in the faeces. Vet. Med. Austria.

[16] Webber J.T., Henley M.D., Pretorius Y., Somers M.J., Ganswindt A. (2018). Changes in African elephant (*Loxodonta africana*) faecal steroid concentrations post-defaecation. Bothalia Afr. Biodivers. Conserv..

